# Sernbo score predicts survival after intracapsular hip fracture in the elderly

**DOI:** 10.1308/003588413X13511609954653

**Published:** 2013-01

**Authors:** EJC Dawe, E Lindisfarne, T Singh, I McFadyen, P Stott

**Affiliations:** Brighton and Sussex University Hospitals NHS Trust,UK

**Keywords:** Hip, Fracture, Mortality, Survival

## Abstract

**Introduction:**

The Sernbo score uses four factors (age, social situation, mobility and mental state) to divide patients into a high-risk and a low-risk group. This study sought to assess the use of the Sernbo score in predicting mortality after an intracapsular hip fracture.

**Methods:**

A total of 259 patients with displaced intracapsular hip fractures were included in the study. Data from prospectively generated databases provided 22 descriptive variables for each patient. These included operative management, blood tests and co-mobidities. Multivariate analysis was used to identify significant predictors of mortality.

**Results:**

The mean patient age was 85 years and the mean follow-up duration was 1.5 years. The one-year survival rate was 92% (±0.03) in the low-risk group and 65% (±0.046) in the high-risk group. Four variables predicted mortality: Sernbo score >15 (*p*=0.0023), blood creatinine (*p*=0.0026), ASA (American Society of Anaesthesiologists) grade >3 (*p*=0.0038) and non-operative treatment (*p*=0.0377). Receiver operating characteristic curve analysis showed the Sernbo score as the only predictor of 30-day mortality (area under curve 0.71 [0.65–0.76]). The score had a sensitivity of 92% and a specificity of 51% for prediction of death at 30 days.

**Conclusions:**

The Sernbo score identifies patients at high risk of death in the 30 days following injury. This very simple score could be used to direct extra early multidisciplinary input to high-risk patients on admission with an intracapsular hip fracture.

Several scoring systems have been used to predict mortality after hip fractures.^1^–4 Most scores rely on the precise definition of co-morbidities and involve formulaic calculation. Information on the co-morbid state of some elderly patients may be difficult to access in the short time before surgery. The Sernbo score is a four-component score that can be calculated easily using information obtained during routine orthopaedic patient assessment.^5^ Patients with hip fractures form a very diverse group of patients from active, independent patients through to extremely frail patients with multiple co-morbidities who are very high-risk surgical candidates. The aim of this study was to assess the role of the Sernbo score in predicting mortality after intracapsular hip fractures as well as to examine its possible role in highlighting patients who might benefit from transfer to a ‘preoperative optimisation area’ for monitoring, correction of fluid and electrolyte abnormalities, and enhanced surgical planning.

## Methods

Patients admitted between December 2009 and January 2011 were identified using prospectively collected hospital databases. All patients over the age of 65 with a displaced intracapsular fracture of the femoral neck were included in the study. Patients were excluded if they had a known malignancy affecting the hip prior to surgery.

All patient records were checked against radiography on the hospital picture archiving and communication system. This allowed verification of the injury pattern and treatment modality.

Information from the National Hip Fracture Database and our local prospective database was used to calculate the Sernbo score for each patient. The Sernbo scoring system is shown in Table 1. This score allows stratification into a high risk and a low-risk group. High-risk patients were defined as those scoring <15 points while low-risk patients scored ≥15 points. The cut-off of 15 points was chosen as this was the threshold described in the original description of the Sernbo score.^5^

Data were collected and verified for 22 variables including operative factors such as grade of surgeon and prosthesis, preoperative blood results and co-morbidity (Table 2). Level of independence and mobility were included in the Sernbo score calculation together with the other two variables of age and mental status.

The data were analysed using MedCalc^®^ version 11.2.1.0 (MedCalc Software, Mariakerke, Belgium). Cox proportional hazards analysis was undertaken, with variables achieving a *p*-value of <0.05 included in the model. Categorical variables were assessed using Fisher’s exact test or a chi-squared test where appropriate. Receiver operating characteristic (ROC) curves were calculated according to the method of DeLong *et al* ^9^ to assess whether the threshold of 15 points was indeed the most appropriate to define the high and low-risk groups. This method was also employed to compare the predictive powers of different variables for death at 30 days. Binomial exact calculation was used to establish confidence intervals for the area under the curve.

**Table 1:** The Sernbo scoring system

Domain		Score
1. Age	<80 years	5
	≥80 years	2
2. Social situation	Independent (no carers)	5
	Package of care / residential care / nursing care	2
3. Mobility	Walks unaided / one stick	5
	Two sticks / frame / wheelchair / bedbound	2
4. Mental state	Normal	5
	History of dementia	2
Total:		/20

**Table 2:** Variables entered into the regression model

Abbreviated mental test score^6^
Age
ASA grade^7^
Cerebrovascular accident
Chronic obstructive pulmonary disease
Clips/sutures used for skin closure
Consultant anaesthetist
Consultant present at surgery
Date of surgery
Diabetes
Ischaemic heart disease
Parkinson’s disease
Postoperative albumin (mmol/l)
Preoperative creatinine (mmol/l)
Preoperative haemoglobin (g/dl)
Preoperative mobility
Preoperative urea (mmol/l)
Prosthesis
Revised cardiac risk index^8^
Sex
Surgeon grade
Time to surgery

**Table 3:** Management of displaced intracapsular fractures by prosthesis

Type	Prosthesis	Frequency
Total hip arthroplasty	Exeter^™^/Trident^®^	37 (14.2%)
	Stanmore	2 (0.77%)
Hemiarthroplasty	Cemented Thompson	125 (47.9%)
	Austin Moore	52 (19.9%)
	Unipolar Exeter^™^	16 (6.1%)
	Charnley–Hastings bipolar	7 (2.3%)
Non-operative management		12 (4.6%)

**Figure 1:**
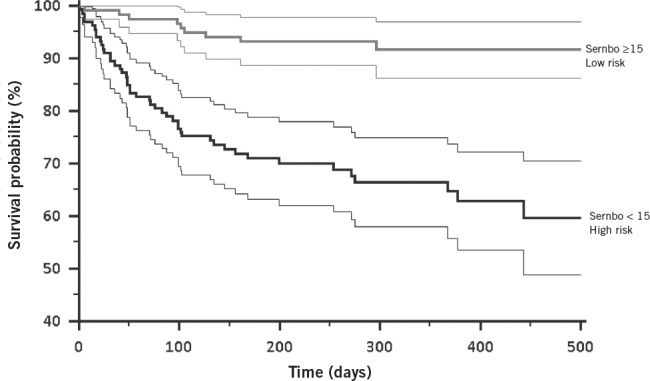
Kaplan–Meier estimate of overall survival

Ethical approval was felt unnecessary for this analysis on routinely collected patient data and was therefore not sought.

## Results

Two hundred and fifty-nine patients sustained a displaced fracture of the femoral neck during the study period. Of these, 56 were male and 203 were female. The mean age was 85 years (range: 66–104 years). The mean follow-up duration was one year and six months. Two patients had sequential bilateral surgery. Patients were managed using either a total hip arthroplasty, a hemiarthroplasty or non-operatively. The choice of management is shown in Table 3.

### Application of the Sernbo score

One hundred and thirty-one patients (51%) scored <15 points on the Sernbo score and were placed in the high-risk group. One hundred and twenty-eight patients (49%) scored ≥15 points and were placed in the low-risk group.

### Survival analysis

The two groups exhibited differing mortality at 30 days (99% survival in the low risk group vs 89% survival in the high-risk group), at 1 year (92% vs 65% survival) and at 1.5 years (92% vs 59%). These results were statistically significant, both by a logrank test of survival curves (*p*<0.0001), and at 30 days (*p*=0.0058), 1 year (*p*<0.0001) and 1.5 years (*p*<0.0001) using a chi-squared test. A Kaplan–Meier estimate of overall survival is shown in Figure 1.

**Table 4:** Covariates included in the Cox proportional hazards regression model

Covariate	Relative risk	95% confidence interval	*P*-value
ASA grade 4	4.89	2.22–10.79	**0.0001**
ASA grade 5	22.89	2.77–188.90	**0.0038**
Non-operative management	3.71	1.08–12.66	**0.0377**
Creatinine (mmol/l)	1.0077	1.00–1.01	**0.0026**
Sernbo score <15	3.845	1.63–9.09	**0.0023**

### Multivariate analysis

Cox proportional hazards analysis produced a statistically significant model (*p*=0.0001) that included 4 of 22 covariates. These were ASA (American Society of Anaesthesiologists) grades 4 or 5, non-operative management and a Sernbo score of <15. The effects of these variables are summarised in Table 4. Date of surgery was not included in the model, showing that there was no time dependent effect on mortality.

### Receiver operating characteristic curve analysis

**Figure 2:**
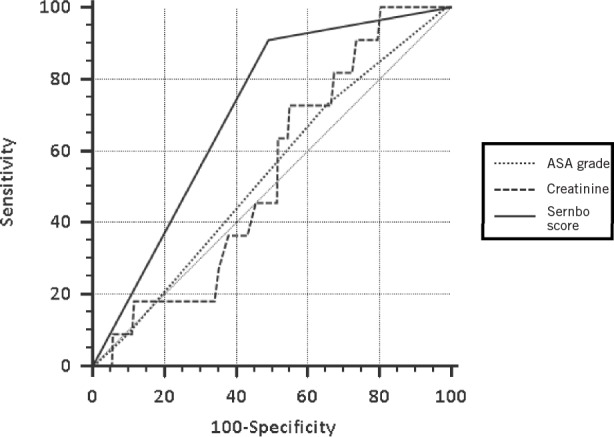
Receiver operating characteristic curves showing the ability of different factors to predict 30-day mortality after hip fracture

ROC curve analysis showed that the cut-off of 15 points used in the original description of the Sernbo score^5^ provided the greatest level of sensitivity and specificity compared with other cut-off values (Fig 2). While the Cox proportional hazards model showed that four variables were predictive of death over the year after hip fracture, ROC curve analysis showed that the Sernbo score was the only factor capable of predicting mortality at 30 days (*p=*0.0001). The area under the curve was 0.71 (0.65–0.76) at 30 days and at 1 year it was 0.68 (0.59–0.75). At 30 days, 1 patient from the low-risk group had died while 12 from the high-risk group had died.

## Discussion

Hip fracture is the most common injury encountered in elderly patients admitted to most trauma services. This burden is expected to increase such that by 2033 there will be 100,000 hip fractures treated each year in the UK.^10^ Risk stratification of patients with a hip fracture may become increasingly important in planning perioperative care, directing management and aiding the process of informed consent.

The Sernbo score divides patients into those with very low mortality (<1% at 30 days) and a high-risk group with much greater mortality (8% at 30 days). The score may be calculated easily by non-specialist staff and could be used to direct high risk patients to a preoperative optimisation area.

### Current strategies for preoptimisation in hip fracture

There is increasing interest in the benefit of providing greater levels of senior multidisciplinary input for patients with hip fractures. A variety of methods are being employed currently to help provide increased anaesthetic, orthopaedic and orthogeriatric input from admission. In some centres, all patients with a hip fracture are reviewed by the on-call anaesthetic team on admission and then again prior to surgery. Other centres use ‘preoperative optimisation’ areas for all patients with neck of femur fractures. This extra input allows optimisation of fluid and electrolyte balance, analgesia, and correction of arrhythmias with the guidance of the anaesthetic and critical care teams. In turn, there may be more effective surgical planning, fewer cancellations in theatre and improved postoperative outcomes.

The provision of this extra input, however, might be unnecessary for many patients in the very diverse group who sustain a hip fracture. There could also be implications for both the cost and resources necessary to provide senior anaesthetic assessment of all patients with a hip fracture on admission.

### Potential for use of the sernbo score in preoperative optimisation

One option for more efficiently directing early critical care or anaesthetic input is to risk stratify patients using a simple non-specialist score and to transfer them to an appropriate area based on the results. The current guidance on hip fracture from the National Institute for Health and Clinical Excellence suggests that surgery should take place on the day or the day after injury.^11^ Any scoring system must therefore be simple to apply in the short time before surgery.

Patients with a Sernbo score of <15 were nearly four times more likely to die over time after a hip fracture than those in the low-risk group (hazard ratio: 3.8). Patients identified as high risk could be managed initially in a preoperative optimisation area with extra senior multidisciplinary input to allow surgical planning. Low-risk patients could be managed on an orthopaedic ward.

High-risk patients often have complex co-morbidities. The presence of dementia or immobility may well make patients unsuitable candidates for critical care interventions as many will lack reversible physiological disturbances. Such patients would benefit from the early involvement of senior orthopaedic, anaesthetic and orthogeriatric staff. This would prevent delays to surgery by the formulation of a coherent surgical strategy for complex patients. Using the Sernbo score to risk stratify patients would also reduce the resources required in a preoperative optimisation area by approximately half compared with a blanket policy.

### The Sernbo score as a method to predict mortality

The Sernbo score was described originally as a method for deciding which patients should receive a hemiarthroplasty and which should receive a total hip arthroplasty after an intracapsular fracture of the femoral neck.^5^ There is, however, a clear rationale behind using these four factors to predict mortality as each is simple to collect and each is an established marker of frailty.

Age has been shown consistently to affect survival after hip fracture.^12^ Our study population had a mean age of 85 years (range: 66–104 years). The monthly risk of death for an 85-year-old woman in the UK is 1 in 141.^13^ In the month after a hip fracture, the risk of a patient in the Sernbo high -risk group increases to 1 in 9 whereas in the low-risk group this increases to only 1 in 100.

Altered mental state has been associated with mortality in hip fracture patients.^14^ The respective roles of dementia and acute confusional state in mortality after hip fractures have been investigated by separate studies. Mortality is in fact more likely to be related to pre-existing dementia^15^ rather than to acute confusional state.^16^ The abbreviated mental test score^6^ does not differentiate well between these conditions but is a routine tool for estimating mental state at admission.

Several studies have examined the association between preinjury residence and postoperative outcome. Residence at a nursing home has been associated with increased mortality.^17,18^ This association has also been extended to all patients who do not live independently.^14,19^ Preinjury mobility also has a well documented association with mortality.^14,18^ This measure is simple to determine from patients, relatives or carers.

None of these factors alone were identified as independent risk factors in our study. This might be due to the inclusion of patients treated non-operatively, who are commonly removed from analyses looking at postoperative survival. To use the Sernbo score on all patients at the time of injury, we included patients who were ultimately treated non-operatively. The strong association with mortality in the non-operative group (relative risk: 3.7) attenuated the ability of the statistical test to pick up other contributing factors in this relatively small sample of 271 fractures.

The rate of non-operative management in this study was slightly higher than expected although this was biased by the study design as only patients over the age of 65 were included in the analysis. The proportion of non-operatively managed patients was at its lowest at the end of the study period and this may reflect a change in practice over time.

### Other scoring systems

Alternative risk stratification tools include the Physiological and Operative Severity Score for the enUmeration of Mortality and morbidity (POSSUM),^2^ the orthopaedic POSSUM,^1^ the Charlson co-morbidity index,^3^ the Estimation of Physiologic Ability and Surgical Stress^20^ and the Nottingham hip fracture score.^4^ Each of these uses a greater number of variables and a greater level of complexity to predict mortality. This information may not be readily available in the short time before surgery. Such scores also require the precise definition of co-morbidities by junior staff. The greatest advantage of the Sernbo score over other existing scores is its simplicity and ease of use by junior and non-specialist staff.

### Confounding variables and study limitations

High ASA grade (grades 3 and 4) and raised preoperative creatinine levels were identified as independent risk factors for mortality in this study. Both creatinine^21^ and ASA grade^18,22^ have been described previously by several studies. Analysis of the effect of the Sernbo score was conducted independently once the effect of ASA grade, creatinine and non-operative management were eliminated in the statistical model. Neither ASA grade nor creatinine were predictive of 30-day mortality. These would not be as useful in identifying patients who might benefit from care in a preoperative optimisation area.

The main limitation of this study is that it was conducted exclusively using patients with intracapsular fractures. Patients with pertrochanteric or subtrochanteric fractures may exhibit a different pattern of mortality after injury. Further work will demonstrate whether this score could be used for patients without an intracapsular fractured neck of the femur.

We were able to identify differences in mortality between the low-risk and high-risk groups using the cut-off of 15 points as suggested in the original description of the score by Sernbo.^5^ One further possibility is that there is excess mortality associated with each increment of the score. This was apparent from our data but did not reach the level of statistical significance. Further studies using larger numbers of patients are needed to investigate this association.

## Conclusions

The Sernbo score is a simple non-specialist tool that may be used as part of a routine orthopaedic assessment to identify high-risk elderly patients prior to surgery for an intracapsular hip fracture. This may have a role as a screening test for transferring patients to a preoperative optimisation area.
